# Antimicrobial resistance in foodborne *Escherichia coli* and *Salmonella* spp. from animal-origin foods: Transmission pathways, global surveillance gaps, and alternative therapeutic strategies

**DOI:** 10.14202/vetworld.2025.3288-3305

**Published:** 2025-11-06

**Authors:** Laura Zhanedilovna Dushayeva

**Affiliations:** Department of Commercialization of Technology and Science, Laboratory of Veterinary and Biological Safety, Zhangir Khan West Kazakhstan Agrarian Technical University, 51 Zhangir Khan, 090009, West Kazakhstan, Uralsk, Republic of Kazakhstan

**Keywords:** animal-origin foods, antimicrobial resistance, antimicrobial stewardship, bacteriophage therapy, *Escherichia coli*, food safety, foodborne pathogens, Kazakhstan, One Health, probiotics, *Salmonella* spp, surveillance systems, sustainable livestock production

## Abstract

Antimicrobial resistance (AMR) in enteric pathogens such as *Escherichia coli* and *Salmonella* spp. has emerged as a critical global health challenge affecting both human and animal populations. The widespread use of antibiotics in food-producing animals for therapeutic, prophylactic, and growth-promoting purposes has accelerated the selection and dissemination of resistant bacteria and resistance genes throughout the food chain. Animal-origin foods, including meat, milk, eggs, and fish, serve as important vehicles for the transmission of multidrug-resistant organisms and AMR genes to humans, representing a significant One Health concern. This review provides an overview of the occurrence, molecular mechanisms, and transmission pathways of AMR in *E. coli* and *Salmonella* isolated from animal-derived foods. Common resistance determinants include β-lactamase genes (*bla_TEM_* and *bla_CTX-M_*), tetracycline resistance genes (*tetA* and *tetB*), and plasmid-mediated quinolone resistance genes, which facilitate horizontal gene transfer through plasmids, integrons, and transposons. Global surveillance reports from World Health Organization’s Global Antimicrobial Resistance Surveillance System, European Food Safety Authority, and World Organization for Animal Health reveal significant regional disparities, with limited monitoring capacity in Central Asia, Africa, and Latin America. Data from Kazakhstan indicate a high prevalence of multidrug-resistant *E. coli* and *Salmonella* in poultry, dairy, and cheese products, underscoring the urgent need for harmonized national surveillance and risk management strategies. The review also discusses alternative approaches to reduce antibiotic use in livestock production, including bacteriophage therapy, probiotics, phytogenic feed additives, vaccination, and nanotechnology-based interventions. While these strategies show promising results in laboratory and pilot studies, their practical application remains constrained by regulatory, economic, and field validation challenges. An integrated One Health strategy, combining surveillance, antimicrobial stewardship, and non-antibiotic interventions, is crucial to mitigating the dissemination of AMR along the farm-to-fork continuum. Strengthening laboratory networks, enhancing data sharing, and promoting collaboration among veterinary, environmental, and public health sectors will be crucial to safeguard food safety and global health security.

## INTRODUCTION

Microorganism-induced foodborne diseases continue to pose a major global public health challenge. The Centers for Disease Control and Prevention identify *Campylobacter*, *Listeria monocytogenes*, *Salmonella*, Shiga toxin-producing *Escherichia coli*, *Shigella*, *Vibrio*, and *Yersinia* as the leading pathogens responsible for foodborne illnesses with the greatest public health impact [1–3]. Among these, *Salmonella* spp. remains a predominant cause of bacterial foodborne infections, accounting for substantial global morbidity and mortality [4, 5]. Each year, *Salmonella* infections are estimated to cause between 200 million and over 1 billion cases worldwide, including approximately 93 million cases of gastroenteritis and around 155,000 deaths [6].

Antimicrobial resistance (AMR) has emerged as a critical public health threat in the 21^st^ century, turning once-treatable foodborne infections into conditions that are increasingly difficult to manage. Resistance may be intrinsic or acquired, with the latter representing a far greater concern. Commensal microorganisms such as *E. coli* and *Staphylococcus aureus* can acquire resistance genes through mobile genetic elements, including plasmids, transposons, and integrons [7, 8]. Although most *E. coli* strains are harmless, pathogenic variants, such as avian pathogenic *E. coli* (APEC), cause significant disease in poultry, leading to major economic losses [9–11].

Global surveillance data from the World Health Organization’s (WHOs) Global Antimicrobial Resistance Surveillance System (GLASS), the European Food Safety Authority (EFSA), and the World Organization for Animal Health (WOAH) reveal widespread multidrug-resistant (MDR) among zoonotic pathogens, particularly against critically important antimicrobials such as fluoroquinolones and third-generation cephalosporins [12, 13]. However, regional surveillance remains limited in areas such as Central Asia, Africa, and Latin America, creating major data gaps [14–16]. Recent studies from Kazakhstan report alarming resistance patterns in both companion animal and foodborne isolates, with *E. coli* strains exhibiting resistance to tetracyclines, doxycycline, fluoroquinolones, and β-lactams, and carrying resistance genes such as *bla*_TEM_, *bla*_OXA_, and tetracycline resistance genes (*tetA* and *tetB*) [17, 18]. In addition, foodborne investigations have identified contamination of cheese with pathogenic *E. coli* O157:H7 and poultry products with MDR *Salmonella* Enterica, posing a serious public health risk [19, 20].

The ongoing evolution of resistant pathogens is outpacing the development of new antibiotics, intensifying the AMR crisis. According to the WHO, the global antibacterial pipeline remains critically limited, with few novel drugs under development [21]. Economic constraints and stewardship obligations have further slowed innovation [22], driving renewed interest in alternative strategies such as bacteriophage therapy, probiotics, and strengthened veterinary antimicrobial stewardship (AMS) programs [23].

Despite growing global attention to AMR, major disparities persist in surveillance coverage, data quality, and the practical implementation of control strategies across regions. Most existing studies focus on high-income countries with robust monitoring systems, while limited information is available from low- and middle-income regions such as Central Asia, Africa, and Latin America. These knowledge gaps hinder accurate assessment of the regional burden, transmission pathways, and molecular mechanisms of resistance in foodborne pathogens. Furthermore, much of the available literature concentrates on clinical isolates, whereas systematic data on AMR in *E. coli* and *Salmonella* spp. from animal-origin foods remain scarce. Kazakhstan and neighboring Central Asian nations, despite being major livestock producers, lack comprehensive, harmonized surveillance programs linking veterinary, food, and environmental sectors. In addition, alternative interventions such as bacteriophage therapy, probiotics, phytogenic additives, and vaccination are underexplored or remain at an experimental stage, with limited regional validation or regulatory frameworks. This collective evidence highlights the urgent need to consolidate global and regional data, assess emerging trends, and evaluate sustainable non-antibiotic solutions within a One Health perspective.

This review aims to provide a comprehensive synthesis of current knowledge on AMR in *E. coli* and *Salmonella* spp. isolated from foods of animal origin, with particular emphasis on molecular mechanisms, transmission routes, and global surveillance systems. It also seeks to identify regional data gaps, especially in Central Asia and Kazakhstan, where systematic monitoring is limited. Furthermore, the review critically evaluates emerging alternative therapeutic and preventive strategies, such as bacteriophage therapy, probiotics, phytogenic feed additives, vaccination, and nanotechnology-based approaches, that could reduce antimicrobial dependence in livestock production. By integrating international evidence with underrepresented regional data, this work aims to support the development of harmonized surveillance, strengthen AMS, and promote One Health–oriented interventions to safeguard food safety and public health.

## SOURCES AND TRANSMISSION MECHANISMS OF AMR PATHOGENS

### Foods of animal origin as sources of AMR pathogens

Foods of animal origin, including meat, poultry, milk, dairy products, eggs, and aquaculture products, are major vehicles for the dissemination of AMR bacteria and resistance genes to humans. Contamination may occur at multiple stages of the production and supply chain, from animal rearing and slaughter to processing, storage, and retail distribution. This farm-to-fork continuum forms a critical component of the One Health AMR challenge, linking agricultural practices directly to public health outcomes [24].

#### Meat and poultry

Among all food commodities, poultry meat represents one of the most frequently reported sources of MDR pathogens. *S. Enterica* and APEC strains isolated from broiler and turkey meat commonly exhibit resistance to fluoroquinolones, β-lactams, and tetracyclines [25]. According to the EFSA, ciprofloxacin resistance in *Salmonella* isolates from broilers exceeded 60% in certain EU countries in 2023, reflecting a narrowing spectrum of effective therapeutic options [26]. In the United States, retail chicken products have been repeatedly associated with MDR *Salmonella* Heidelberg and extended-spectrum β-lactamase (ESBL)-producing *E. coli* [27]. Collectively, these observations highlight poultry meat as a central reservoir of resistant enteric bacteria within the global food chain.

#### Milk and dairy products

Raw milk and unpasteurized dairy products are recognized reservoirs of resistant bacteria, including ESBL-producing *E. coli* and methicillin-resistant *S. aureus*. These isolates often demonstrate resistance to critical antimicrobials such as third-generation cephalosporins [28]. In Central Asia, Kuzeubayeva *et al*. [29] reported that approximately 31% of cheese samples were contaminated with *E. coli*, including the O157:H7 serotype, which exhibits resistance to β-lactams, sulfonamides, and quinolones. Such findings reveal both a substantial food safety risk and the limited extent of regional surveillance systems, emphasizing the need for stronger regulatory frameworks to monitor raw dairy products.

#### Eggs

*Salmonella* Enteritidis remains the predominant AMR pathogen associated with eggs and egg-based products. Its persistence in the food chain is driven by both vertical transmission (from infected hens to eggs) and horizontal contamination (during collection, handling, and storage) [30]. Resistant isolates frequently exhibit resistance to quinolones and sulfonamides, complicating outbreak control and treatment [31]. These findings underscore the importance of on-farm biosecurity, regular screening, and effective hygiene management in preventing pathogen persistence and transmission.

#### Fish and aquaculture

The aquaculture industry is increasingly recognized as a potential hotspot for AMR emergence due to the prophylactic and therapeutic use of antibiotics in fish farming. MDR *Aeromonas* and *Vibrio* species have been detected in fish and shellfish destined for human consumption, often carrying transferable resistance genes such as *tet*, *sul*, and *qnr* [32]. An overview of key food sources, associated AMR pathogens, and resistance profiles is summarized in Table 1. A systematic review identified Asia and Latin America as regions most affected by AMR in aquaculture products, though corresponding data from Central Asia remain limited [33]. These findings suggest that, without stringent antimicrobial regulation and environmental monitoring, aquaculture could become a major contributor to AMR dissemination in aquatic and terrestrial ecosystems.

**Table 1:** Animal origin foods as sources of AMR pathogens.

Food product	Main pathogens	Resistance profile/genes	Region (examples)
Poultry meat	*Salmonella* Enterica, APEC	Fluoroquinolones, b-lactams, tetracyclines	EU, USA, and Asia
Milk	*ESBL-Escherichia coli* and MRSA	*bla*_TEM_, *bla*_CTX-M_, and *mecA*	Central Asia, Africa, and the EU
Eggs	*Salmonella* Enteritidis	Quinolones, sulfonamides	EU, Asia
Fish and aquaculture	*Aeromonas and Vibrio* spp.	tet, sub, and qnr genes	Asia and Latin America

APEC = Avian pathogenic *Escherichia coli,* MRSA = Methicillin-resistant *Staphylococcus aureus*, EU = European Union, USA = United States of America

#### Synthesis

Overall, foods of animal origin represent significant sources of resistant bacteria and resistance genes circulating through the food supply chain. Yet, systematic and harmonized surveillance remains limited in many regions, particularly in Central Asia, Africa, and South America, creating substantial blind spots in the global AMR landscape. Addressing these disparities through coordinated monitoring, comparative research, and One Health–based interventions is essential to enhance food safety, protect public health, and mitigate the global spread of AMR.

### Routes of AMR pathogen transmission

The transmission of AMR from food-producing animals to humans occurs through a complex, multilayered pathway that spans from primary production at farms to international food trade. Understanding these interconnected “routes of transmission” is essential for accurately assessing public health risks and developing effective One Health interventions.

#### Farm level

At the production stage, the widespread and often indiscriminate use of antimicrobials for therapeutic, prophylactic, and growth-promoting purposes exerts strong selective pressure on microbial populations. This pressure leads to the emergence and proliferation of resistant bacteria, including *E. coli* [34–38] and *Salmonella* spp., which can colonize animal intestines, persist in the gut microbiota, and be excreted through feces [39]. These resistant organisms then serve as primary sources of environmental contamination and potential zoonotic transmission.

#### Environmental level

The environment serves as a critical secondary reservoir and conduit for the dissemination of resistance. Resistant bacteria and antimicrobial resistance genes (ARGs) are spread through animal manure, wastewater effluents, and contaminated soil, facilitating horizontal gene transfer among diverse microbial communities [40]. This environmental contamination increases the risk of resistance transmission to crops, wildlife, and surface or groundwater systems, reinforcing the cyclical nature of AMR spread between animals, humans, and ecosystems.

#### Food chain level

The food production and processing continuum represents the most direct route of human exposure. Resistant bacteria can contaminate raw meat, milk, eggs, and other animal-derived foods during slaughter, processing, handling, and storage [41]. Inadequate hygiene practices, improper cooking, and cross-contamination in domestic or commercial kitchens further magnify the risk of human infection. Thus, food acts not only as a vehicle for resistant pathogens but also as a bridge linking agricultural practices to human health outcomes.

#### Trade and market level

At the trade stage, both national and international food markets serve as amplifiers for the dissemination of AMR. The globalized trade of animal-origin foods facilitates the transboundary movement of resistant bacteria and their mobile genetic elements, complicating surveillance and containment efforts [42]. In many low- and middle-income countries, informal and poorly regulated trade networks exacerbate the spread by bypassing established food safety and monitoring systems.

#### Integration and control points

Critical control points exist at each stage of the farm–environment–food–trade continuum. These include excessive antimicrobial use (AMU) at the farm level, inadequate waste management in the environment, insufficient hygiene and biosecurity measures in food processing, and fragmented monitoring at the trade level. Addressing these vulnerabilities demands coordinated action, integrating surveillance, policy, and capacity building, across agriculture, public health, and trade sectors under the One Health framework.

**Figure 1:**
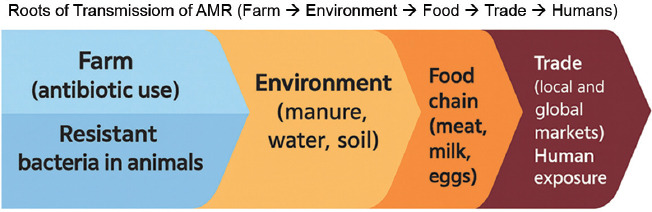
Sankey diagram illustrating the roots of antimicrobial resistance transmission from the farm to humans through the environment, food chain, and trade.

In the following section, a Sankey diagram is presented to visualize the interconnected pathways of antimicrobial resistance (AMR) transmission across the farm–environment–food–trade continuum (Figure 1). The diagram illustrates the multidirectional flow of resistance determinants between sectors, emphasizing their complex interdependence. Despite growing evidence of cross-sectoral AMR dissemination, comparative analyses across regions remain limited. Notably, data from Central Asia, Africa, and Latin America are still underrepresented, hindering the establishment of reliable global baselines. These gaps underscore the urgent need for harmonized, integrated surveillance systems that capture data from the farm, environment, and food chain within the One Health framework.

Despite increasing evidence on these transmission pathways, systematic interregional comparisons remain limited. Data from Central Asia, Africa, and Latin America are particularly scarce, impeding the establishment of global baselines for AMR risk assessment. Therefore, harmonized surveillance systems that integrate farm, environmental, and food chain data are urgently needed to enable early detection, targeted intervention, and effective global risk management.

### AMR mechanisms and global surveillance data

#### AMR transmission pathways in the food chain: A farm-to-fork perspective

**Figure 2:**
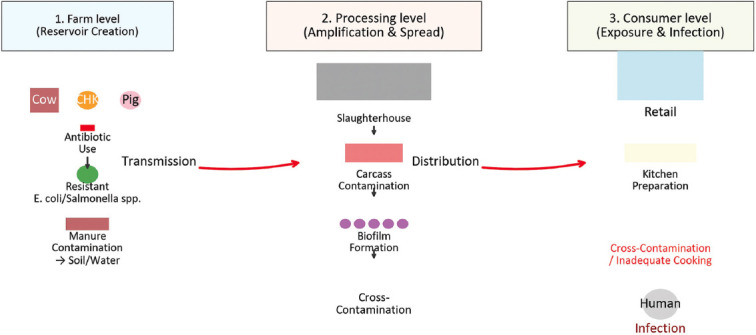
Farm-to-fork transmission pathways of antimicrobial resistance (AMR). The three critical levels of AMR transmission are represented schematically: (1) The creation of resistant reservoirs at the farm level, (2) the amplification and spread during slaughter and processing, and (3) the final exposure and potential infection at the consumer level. This framework provides a visual foundation for the subsequent detailed analysis of the key pathogens.

The dissemination of AMR from food-producing animals to humans is a complex, multi-stage process occurring along the entire food production continuum. Understanding these pathways is essential for identifying intervention points and developing effective control strategies under the One Health framework. The “farm-to-fork” model (Figure 2) illustrates how resistant bacteria and their genes move through this continuum, from farm environments to consumers.

#### Reservoir formation at the farm level

The farm serves as the initial reservoir for the development of resistance. Extensive AMU for therapy, prophylaxis, and growth promotion applies strong selective pressure on the gut microbiota of animals, promoting the enrichment of resistant bacteria such as *E. coli* and *Salmonella* spp. [43]. These bacteria are shed profusely in feces, contaminating soil, water, and farm equipment, thereby establishing a persistent environmental reservoir that perpetuates re-exposure and further transmission.

#### Amplification and spread during slaughter and processing

Slaughterhouses represent critical control points where contamination is frequently amplified. During evisceration, intestinal contents containing resistant bacteria can spread to carcasses, equipment, and processing surfaces. Resistant *E. coli* and *Salmonella* readily form biofilms on conveyor belts, knives, and drains, allowing them to persist despite disinfection efforts [44]. These biofilms act as continuous sources of contamination, making the slaughter and processing stages among the weakest links in the food safety chain.

#### Exposure at retail and consumer levels

Once contaminated products reach the retail level, consumers face direct exposure risks. Inadequate refrigeration, prolonged storage, and poor handling can facilitate bacterial proliferation. Cross-contamination, such as using the same utensils or cutting boards for raw meat and ready-to-eat foods, further increases the likelihood of transmission. Insufficient cooking temperatures may fail to eliminate resistant pathogens, resulting in colonization or infection [45].

This holistic “farm-to-fork” perspective underscores that AMR is not merely a microbiological issue but a systems-level problem spanning agriculture, industry, and households. Preventive control measures must be implemented at every stage, biosecurity at farms, sanitary practices during slaughter and processing, and food hygiene at the consumer level. However, failures at any point can undermine progress elsewhere.

Global surveillance programs such as WHO GLASS, EFSA/European Centre for Disease Prevention and Control (ECDC), and WOAH consistently recognize these transmission routes, though reporting quality and data completeness vary widely, particularly in low- and middle-income countries (LMICs), including Kazakhstan. This emphasizes the urgent need for harmonized surveillance to capture the full dynamics of AMR transmission across the food chain.

#### Global AMR surveillance systems and data gaps

Effective surveillance is fundamental to understanding and mitigating AMR within the food chain. Several international frameworks exist, yet their scope, coverage, and implementation remain highly uneven.

The WHO’s GLASS provides a standardized platform for collecting and sharing AMR data across countries. Initially focused on human pathogens, GLASS has progressively expanded to include foodborne bacteria, enabling integrated One Health analyses. However, participation is voluntary, and LMIC representation remains limited [46].

The WOAH requires member countries to report AMU and AMR data in animals through its annual global database. WOAH guidelines emphasize harmonized monitoring of key indicator organisms, particularly *E. coli* and *Salmonella* spp. [47]. Nonetheless, data quality and compliance vary considerably, constrained by weak veterinary laboratory infrastructure in many regions.

The European Union operates the most comprehensive regional AMR monitoring framework through the EFSA and the ECDC. These agencies jointly publish annual reports that integrate AMR data from humans, animals, and food, providing a robust basis for policymaking [48]. However, the system’s geographic limitation to EU member states leaves substantial global blind spots.

#### Persistent surveillance gaps

Major data deficiencies continue to hinder global AMR understanding:

- Lack of standardized sampling protocols and laboratory capacity in LMICs;
- Absence of integrated databases linking human, veterinary, and environmental surveillance;
- Limited monitoring of emerging or rare resistance mechanisms;
- Weak coordination between public health and veterinary authorities.

These disparities fragment the global picture of AMR in the food chain, leading to an underestimation of the true burden and slowing international response efforts. Establishing harmonized, interoperable surveillance systems remains a top global priority for achieving One Health-aligned AMR management.

#### E. coli in the food chain: Mobilizing resistance from farm to fork

*E. coli*, a ubiquitous commensal in humans and animals, serves as both a sentinel and a genetic reservoir for ARGs across the food production ecosystem. Its remarkable capacity for horizontal gene transfer makes it a key driver of AMR dissemination across species, environments, and geographic boundaries.

#### Global burden and transmission dynamics

The intensive use of antimicrobials in livestock production has led to worldwide. Resistance patterns reflect regional antimicrobial usage, with tetracycline resistance, mediated by efflux pump genes such as *tet(A)*, being particularly prevalent in isolates from poultry, swine, and cattle [49]. Current surveillance often overlooks the diversity of resistance determinants, as many isolates lacking common *tet* genes still display phenotypic resistance, implying unrecognized variants such as *tet(M)* and *tet(G)* [50].

Beyond tetracyclines, *E. coli* from animal sources frequently harbor plasmid-mediated quinolone resistance genes (*qnrS*) and ESBLs such as *bla*_CTX-M_ and *bla*_TEM_ [51]. These elements drive rapid horizontal spread of reduced susceptibility to fluoroquinolones and cephalosporins, establishing MDR profiles that permeate the food chain.

#### Mobile genetic elements: Engines of dissemination

The global propagation of ARGs is largely mediated by mobile genetic elements (MGEs), including plasmids, integrons (e.g., *Int1*), and insertion sequences (e.g., *IS3*) [52]. These genetic vehicles enable the assembly and transfer of multi-resistance cassettes across bacterial populations. Identical resistance genes, such as *bla*_TEM-163_, *aph(3’)-Ib*, and *qacE*, have been identified in *E. coli* isolates from human, animal, and environmental sources across more than 70 countries [53].

High-income agricultural nations, such as China and the United States, exhibit the greatest ARG diversity, reflecting both historical and ongoing antimicrobial selection pressures [54]. The co-localization of β-lactamase genes (*bla*_CTX-M_ and *bla*_TEM_), tetracycline determinants (*tetA*), and *qnrS* on conjugative plasmids accelerates co-selection, stabilizing MDR phenotypes across global *E. coli* populations.

#### Biofilms and environmental persistence

**Figure 3:**
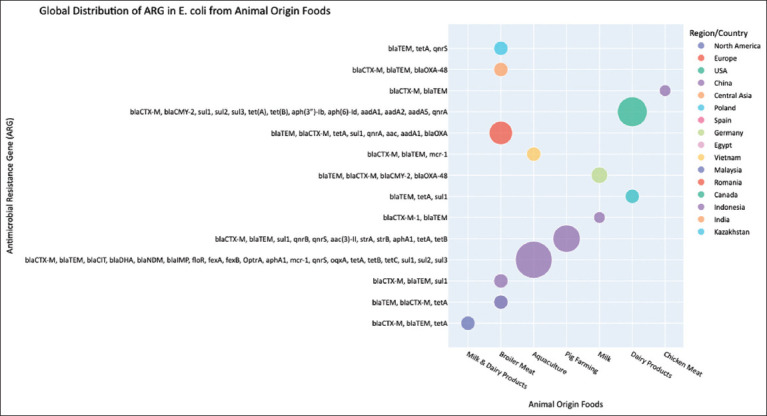
Global distribution of antimicrobial resistance genes in *Escherichia coli* from animal origin foods [59–70].

A critical factor enhancing *E. coli*’s persistence is its ability to form biofilms, structured microbial communities adhering to surfaces throughout the food chain, from farm equipment to food processing facilities [55]. Biofilm-associated bacteria exhibit heightened tolerance to disinfectants and antibiotics, allowing resistant strains to endure sanitation procedures. Ampicillin- and tetracycline-resistant strains often display superior biofilm-forming capacity [56], with fimbriae-mediated adhesion enhancing resistance and colonization potential, particularly in ESBL-producing isolates [57]. These biofilms act as reservoirs that perpetuate contamination cycles and complicate eradication efforts, leading to infections that are difficult to treat, such as bloodstream or urinary tract infections in humans [58]. The global distribution of antimicrobial resistance genes in *E. coli* from animal-origin foods is shown in Figure 3 [59–70].

#### Global patterns and surveillance insights

A synthesis of global data (Figure 3) reveals distinct regional variations in ARG prevalence among *E. coli* isolates from animal-derived foods [59–70].

- In dairy products, isolates from North America, Central Asia, and Europe predominantly carry β-lactamase genes (*bla*_CTX-M_, *bla*_TEM_, and *bla*_CMY-2_) alongside *tet(A/B)*, and *sul1–3* determinants, consistent with widespread ESBL and AmpC phenotypes
- Broiler meat from Europe, the USA, and Asia shows consistently high MDR frequencies, with *bla*_CTX-M_, *bla*_TEM,_ *tetA*, and *qnrA/S* commonly detected
- Aquaculture samples from Asia frequently harbor mobile ARGs conferring resistance to β-lactams, tetracyclines, sulfonamides, and even colistin (*mcr-1*), reflecting the intensive use of antibiotics in fish farming.

These findings demonstrate that AMR dissemination in *E. coli* follows region-specific patterns influenced by local AMU practices. However, fragmented surveillance in LMICs prevents comprehensive global assessments and hinders targeted interventions.

#### Public health implications

The persistence and spread of resistant *E. coli* of animal origin through food and water pose a significant global health burden. These strains are increasingly responsible for bloodstream and intra-abdominal infections, often associated with treatment failure [71]. Fluoroquinolone prophylaxis failures in hematology patients exemplify the clinical repercussions of AMR selection within animal reservoirs [72].

The food chain thus serves as a critical transmission route, not only for resistant organisms but also for the mobile genetic elements that drive their spread. Addressing this challenge requires transboundary, integrated surveillance systems that monitor the flow of resistant clones and ARGs across the human–animal–environment interface, forming the foundation for effective One Health AMR mitigation strategies.

#### Salmonella spp. in the food chain: A global overview of AMR threats and transmission routes

**Figure 4:**
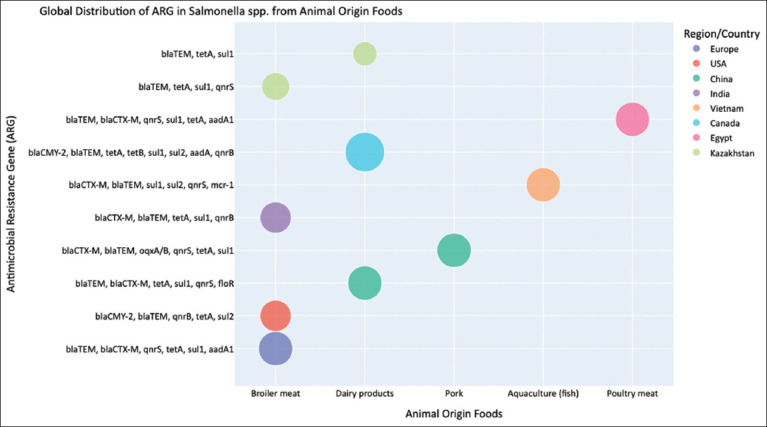
Global distribution (2021–2025 years) of *Salmonella* spp. in foods of animal origin [8, 12, 15, 17, 20, 22, 39, 41, 44, 54, 55].

*Salmonella* spp. remain among the most important bacterial causes of foodborne disease worldwide, responsible for an estimated 150 million cases and approximately 60,000 deaths annually. Human infection typically results from the consumption of contaminated animal-origin foods, with poultry meat and eggs serving as the dominant sources in most regions [73]. This epidemiological pattern is consistent globally and exemplifies *Salmonella* as a quintessential One Health pathogen. The globalization of food animal trade has further facilitated the international dissemination of successful, MDRclones, such as *Salmonella* Enteritidis and *Salmonella* Typhimurium, transforming localized AMR issues into global public health concerns [74]. The global distribution of *Salmonella* spp. in foods of animal origin from 2021 to 2025 is shown in Figure 4 [8, 12, 15, 17, 20, 22, 39, 41, 44, 54, 55].

#### National context: The case of Kazakhstan

The situation in the Republic of Kazakhstan highlights both national progress and ongoing challenges. A significant reduction in salmonellosis incidence has been observed, from 8,827 reported cases in 1990 to 498 in 2021, yet the epidemiological profile warrants attention. *Salmonella* Enteritidis remains the predominant serovar, accounting for 76.4%–88.4% of cases, followed by *Salmonella* Typhimurium. Notably, an increasing proportion of cases now occur among children under 14 years of age [44, 45]. Between 70% and 90% of infections are foodborne, with poultry meat and eggs implicated in 35%–60% of cases. This underscores poultry production as a critical control point in the national farm-to-fork continuum, mirroring global evidence that identifies contaminated poultry as the principal vehicle for human salmonellosis [75].

#### Drivers of resistance and global dissemination

Poultry farming, a cornerstone of global food production, also serves as a major reservoir for AMR *Salmonella* spp. The widespread use of antimicrobials for prophylaxis, therapy, and growth promotion exerts strong selective pressure, favoring the development of resistant strains. A study from broiler farms in Malaysia (2018–2019) reported MDR in 82% of *Salmonella* isolates, a trend echoed in Southeast Asia, Africa, and the Americas, demonstrating that antimicrobial misuse in animal production is a universal driver of resistance [76, 77].

*Salmonella* spp. exhibits a remarkable ability to acquire and disseminate resistance genes through MGEs such as plasmids and transposons. Chromosomal mutations in *gyrA* and *parC* confer fluoroquinolone resistance, while plasmid-mediated genes, including *bla*_TEM_ (ampicillin resistance), *tet(A/B)* (tetracycline resistance), and *qnr* (quinolone resistance), are frequently detected in isolates from meat and environmental samples [78, 79]. These MGEs enable horizontal gene transfer between bacterial species within animal intestines, during food processing, and even within food matrices. Consequently, the food chain acts not only as a conduit for resistant pathogens but also as a reservoir of transferable resistance genes.

#### Serovar-specific resistance trends

The prevalence of MDR *Salmonella* serovars varies geographically, reflecting regional AMU practices and surveillance capabilities:

- *Salmonella* Enteritidis, traditionally considered more susceptible, now exhibits growing resistance, particularly to nalidixic acid and fluoroquinolones, in poultry-derived isolates from multiple countries [80].
- *Salmonella* Typhimurium, especially its penta-resistant DT104 clone (ACSSuT phenotype), remains strongly associated with MDR profiles in pork, beef, and poultry worldwide [81].
- *Salmonella Infantis* has emerged as a dominant MDR serovar in poultry production across Europe, the Americas, and the Middle East, often harboring large plasmids carrying multiple resistance genes.
- *S. Heidelberg*, prevalent in North American poultry and retail meats, frequently shows resistance to extended-spectrum cephalosporins and fluoroquinolones [82].

These shifting serovar patterns and escalating resistance profiles reflect not only microbial adaptation but also agricultural practices that perpetuate selection pressure, transforming AMR in *Salmonella* into a biological indicator of antimicrobial misuse.

#### Knowledge gaps and surveillance needs

Despite advancements, major knowledge gaps limit a full understanding of AMR in *Salmonella* within the global food system.

- Surveillance disparities: Many low- and middle-income countries lack harmonized AMR monitoring programs in food-producing animals and retail meats, leading to underreporting of emerging MDR clones
- Transmission dynamics: The role of environmental routes, such as contaminated irrigation water, soil, and non-poultry food sources, remains poorly characterized
- Intervention strategies: The effectiveness of alternative control measures, including bacteriophage therapy, vaccination, and AMS initiatives, requires further field-based validation.

#### Synthesis

Controlling *Salmonella* in the food chain extends beyond eradicating a single pathogen, it involves disrupting a dynamic reservoir of resistance genes capable of crossing ecological and geographic boundaries. Addressing this global AMR threat demands harmonized, transboundary surveillance systems capable of tracking resistant clones and MGEs from farm to consumer. Integrating genomic surveillance, responsible antimicrobial policies, and One Health-based interventions is essential to mitigate the growing public health impact of foodborne *Salmonella* infections.

## OVERVIEW OF AMR DETECTION, SURVEILLANCE, AND CONTROL STRATEGIES

### AMR detection and surveillance methods

Effective AMR surveillance relies on a combination of phenotypic and genotypic approaches to identify resistant pathogens and track emerging trends across the food chain. Phenotypic methods, including culture-based assays such as disk diffusion and broth microdilution, as well as automated systems such as VITEK2 and BD Phoenix, remain the gold standard for antimicrobial susceptibility testing (AST). These techniques measure the minimum inhibitory concentration, providing critical insights into the actual resistance profiles of pathogens such as *E. coli* and *Salmonella* spp. Although reliable, they are time-intensive and often limited by throughput constraints.

Genotypic methods, including polymerase chain reaction and whole-genome sequencing (WGS), have revolutionized AMR surveillance by enabling the rapid detection of specific ARGs directly from isolates or environmental samples. Among these, WGS has emerged as a transformative tool, offering high-resolution data for identifying resistance mechanisms, tracking outbreak strains, and reconstructing transmission dynamics along the farm-to-fork continuum [83]. The integration of phenotypic and genotypic methods within global and national programs, such as WHO GLASS and EFSA/ECDC surveillance frameworks, is essential for generating comparable, evidence-based datasets on AMR trends in zoonotic pathogens originating from animal-derived foods.

### Alternative therapies and stewardship strategies for AMR control

#### Expanding the arsenal beyond antibiotics

The declining efficacy of conventional antibiotics has intensified efforts to develop alternative, non-antibiotic strategies to control AMR in foodborne pathogens. A multi-pronged approach, combining direct antimicrobial interventions with strong stewardship principles, is vital to preserve the effectiveness of existing drugs [84].

Bacteriophage therapy has demonstrated encouraging results in reducing *Salmonella* and *E. coli* colonization in poultry and livestock, with successful field applications in countries such as Georgia and Poland. In Kazakhstan, phage research remains in the experimental phase, underscoring the gap between scientific potential and practical implementation. Establishing national phage banks and developing regulatory frameworks are key prerequisites for advancing these technologies into veterinary practice.

Probiotics, prebiotics, synbiotics, and phytogenic feed additives are also being investigated to enhance gut health, modulate microbiota, and suppress colonization by resistant bacteria. Large-scale adoption in China and India has demonstrated measurable success; however, in Kazakhstan, application remains limited to commercial formulations with minimal adaptation to local microbial ecosystems. Similarly, phytotherapy, using regionally available medicinal plants, offers an affordable and accessible option, though its efficacy under field conditions remains largely unvalidated. Despite their potential, these approaches face economic, regulatory, and awareness barriers, emphasizing the need for context-specific, evidence-driven strategies to achieve sustainable implementation.

#### Comparative analysis of non-antibiotic therapies

Non-antibiotic interventions differ in mechanisms, efficacy, and scalability:

- Bacteriophage therapy: Offers high specificity, targeting pathogenic bacteria while sparing beneficial flora. Phages can coevolve with their hosts, thereby minimizing the development of resistance [85, 86]. However, challenges include narrow host ranges, regulatory uncertainty, and standardization of production protocols [87]. Although approved for use in food decontamination against *Listeria* spp. and *Salmonella* spp, in some countries [88], adoption in Central Asia remains limited due to the absence of national regulatory frameworks and phage repositories
- Microbiome modulation: Probiotics, prebiotics, and synbiotics reduce pathogen carriage by enhancing gut health. Meta-analyses report up to 45% reductions in *Salmonella* prevalence and decreased shedding of resistant *E. coli* in poultry [89]. Nevertheless, their efficacy depends on strain selection, host species, and management conditions, emphasizing the need for locally tailored formulations [90, 91]
- Vaccination, antimicrobial peptides (AMPs), and nanotechnology: Vaccines reduce infection incidence and antibiotic demand, while AMPs, essential oils, organic acids, and nanocarriers offer novel delivery systems and synergistic antimicrobial effects. However, field-level data remain sparse, particularly in LMICs such as Kazakhstan, limiting evidence-based policymaking.

These approaches vary in mechanisms, efficacy, and scalability (Table 2 shows a comparative summary of non-antibiotic therapies, their mechanisms, global applications, and challenges) [92–100].

**Table 2:** A comparative overview of alternative approaches to combat AMR (global perspective) [92–100].

Alternative strategy	Mechanism of action	Global status	Implementation challenges
Bacteriophage therapy	Targeted lysis of pathogenic bacteria (*Salmonella* and *Escherichia coli*)	Applied in poultry and food safety in Georgia, Poland, USA; commercial products approved for food decontamination	Regulatory uncertainty, narrow host range, and the need for phage banks
Probiotics, prebiotics, and synbiotics	Modulation of the gut microbiota and competitive exclusion	Widely used in poultry/swine farming in China, India, and the European Union	Strain specificity and variable efficacy
Phytogenic feed additives	Plant-derived antimicrobial and immunomodulatory compounds	Expanding use of LMICs in Asia	Non-standardized formulations and variable bioavailability
Vaccination	Prevention of *Salmonella* infection and other infections	Implemented in the EU, USA, and Asia	High cost, limited coverage, and cold chain
AMPs and nanotechnology	Broad-spectrum antibacterial activity and targeted delivery	Global experimental stage	High cost, scalability, and safety concerns

AMR = Antimicrobial resistance, AMPs = Antimicrobial peptides, LMICs = Low- and middle-income countries.

Collectively, these alternatives offer a diverse toolbox for reducing antimicrobial dependence in livestock systems. While probiotics, phytogenic additives, and vaccination are gaining traction, phage and nanotechnology-based approaches remain largely experimental. Key barriers include cost, variable efficacy, and weak regulatory capacity, particularly in developing economies. Overcoming these challenges requires targeted research, regional adaptation, and strengthened policy support.

Although phage therapy holds great promise, its deployment in livestock is limited by host specificity, production scalability, regulatory ambiguity, and potential immune interference. Optimizing phage cocktails, developing standardized manufacturing protocols, and integrating regulatory oversight are necessary steps to enable field adoption and measurable impact on AMR reduction.

Overall, non-antibiotic therapies should be viewed not as replacements but as complementary tools that strengthen AMS frameworks. Their successful integration requires coordinated efforts among scientists, veterinarians, policymakers, and producers to translate laboratory innovations into sustainable agricultural practices.

#### Integrating AMS with alternative therapies

AMS remains a cornerstone in global efforts to mitigate AMR. In veterinary and food animal production, AMS promotes responsible antibiotic use through veterinary oversight, evidence-based prescribing, and policy restrictions on growth promoters [101, 102]. However, stewardship alone cannot succeed without complementary interventions that reduce infection pressure and antibiotic demand.

Integrating alternative therapies, such as phages, probiotics, prebiotics, and vaccines, within AMS frameworks can significantly reduce antibiotic reliance across the production chain. Phages provide pathogen-specific biocontrol, probiotics enhance gut resilience, and vaccines prevent infections, thereby reducing AMU [103, 104]. Together, these tools create a multi-layered defense against resistant pathogens.

Nonetheless, adoption remains constrained by cost, regulatory complexity, and variable efficacy across species and production systems. In addition, most alternatives lack validation under field conditions. These challenges reinforce the notion that no single approach can replace antibiotics entirely; instead, a synergistic, One Health-based strategy is essential [105].

To achieve this, countries must:

- Strengthen integrated surveillance aligned with WHO GLASS and WOAH standards;
- Develop economic incentives for the production and validation of veterinary alternatives;
- Harmonize regulatory pathways for approval and commercialization;
- Promote farmer education and capacity building to ensure correct application.

In Kazakhstan and other LMICs, where AMR initiatives remain at the strategic planning stage, practical implementation will depend on localized research, region-specific product development, and policy support.

Ultimately, alternative therapies must be embedded within robust AMS programs under the One Health framework, ensuring science-driven validation, cross-sector collaboration, and sustainable reduction in antibiotic use in food-producing animals. Aligning innovation with stewardship offers the most viable pathway toward curbing the global AMR crisis.

## CHALLENGES AND FUTURE DIRECTIONS IN AMR CONTROL

### Global burden and One Health significance

AMR in foodborne pathogens such as *E. coli* and *Salmonella* spp. poses a critical threat to human and animal health worldwide. Foods of animal origin – particularly meat, milk, eggs, and fish – serve as important reservoirs and transmission routes for resistant bacteria along the farm-to-fork continuum. Factors including inappropriate AMU on farms, poor biosecurity and hygiene during slaughter and processing, and improper handling or cooking at the consumer level all contribute to the dissemination of resistant organisms [106, 107].

Despite its recognized importance, surveillance data remain incomplete across many regions, limiting the capacity to assess transmission dynamics and guide evidence-based interventions. This gap underscores the urgent need for integrated, cross-sectoral monitoring within the One Health framework, linking veterinary, food, environmental, and human health systems.

### Regional disparities in AMR surveillance and control

A comparative analysis of global experiences reveals marked disparities in AMR management between high-income and low- to middle-income regions.

In high-income countries, including the European Union, the United States, and Oceania, harmonized surveillance, strict veterinary drug regulations, and targeted AMS programs have resulted in measurable declines in AMR prevalence in livestock-derived foods [108, 109].

In contrast, many low- and middle-income countries (LMICs) continue to face major challenges, including unregulated antimicrobial sales, inadequate diagnostic infrastructure, and insufficient monitoring of animal-origin foods.

In Central Asia, countries such as Kazakhstan are making progress by strengthening laboratory capacity and aligning national policies with international frameworks through collaborations with the WHO and the Food and Agriculture Organization. However, surveillance systems remain fragmented and underdeveloped relative to those in Europe or North America [110, 111]. These disparities underscore the need for sustained investment in laboratory infrastructure, capacity building, and effective policy enforcement to ensure the containment of AMR.

### Economic implications of AMR in livestock production

Beyond public health concerns, AMR in food animals imposes substantial economic burdens. The associated costs include increased veterinary expenditures, reduced productivity due to disease-related morbidity and mortality, and potential trade restrictions on contaminated or non-compliant animal products. These factors collectively threaten food security and international market access. While an in-depth economic evaluation was beyond the scope of this study, future research should integrate economic impact analyses to inform policy design, prioritize resource allocation, and support sustainable livestock production systems.

### Limitations in One Health integration and data sharing

A key limitation in global AMR control efforts is the limited integration of data across human, animal, and environmental sectors. Although frameworks such as WHO GLASS, EFSA/ECDC joint monitoring, and WOAH reporting systems have standardized surveillance practices, the implementation of the One Health approach remains inconsistent. This fragmentation reduces the effectiveness of national AMR action plans and hinders coordinated responses across sectors [94, 95].

Addressing these gaps requires the harmonization of data collection methods, interoperable databases, and shared risk assessment tools to achieve comprehensive, real-time AMR monitoring [97].

### Advances and challenges in alternative therapeutic approaches

The development of non-antibiotic interventions represents an important complementary strategy in AMR control.

Bacteriophage therapy has demonstrated encouraging results in reducing *E. coli* and *Salmonella* infections in both clinical and agricultural settings, particularly in Georgia, Poland, and Russia [112, 113]. However, its widespread application remains constrained by several challenges, including narrow host range, variable efficacy under field conditions, host immune interference, and the absence of standardized production and regulatory frameworks. Overcoming these limitations through optimized phage cocktails, robust manufacturing protocols, and policy development will be essential for transitioning phage therapy from experimental use to practical veterinary application.

Other approaches, such as probiotics, prebiotics, competitive exclusion cultures, and vaccination, have also shown promise in mitigating AMR transmission through the food chain. Nevertheless, their adoption in veterinary practice remains limited due to insufficient field validation, inconsistent product quality, and a lack of farmer awareness [114, 115].

### Role of innovative diagnostics and surveillance tools

Emerging diagnostic technologies, including rapid molecular assays, point-of-care tests, and WGS platforms, have the potential to transform AMR detection and guide precision AMU. These tools enable faster diagnosis, improved traceability, and targeted interventions, thereby reducing unnecessary antibiotic exposure [116–118]. However, their high cost and technical demands restrict accessibility in many LMICs. Expanding access to affordable diagnostic technologies should therefore be prioritized within national AMR strategies to strengthen early detection and response capacity.

### Future directions and one health policy integration

Current evidence suggests that progress in mitigating AMR remains uneven across regions, reflecting differences in surveillance maturity, policy enforcement, and the adoption of alternative interventions. Effective AMR control requires synergistic alignment between surveillance, regulation, and innovation, addressing not only microbial resistance mechanisms but also the broader socio-economic and ecological determinants of AMR transmission through animal-origin foods.

The integration of AMS, innovative diagnostics, and validated non-antibiotic therapies within a One Health framework is essential to achieve sustainable AMR mitigation. Strengthening transboundary cooperation, harmonizing regulatory standards, and fostering local research capacity, particularly in LMICs such as Kazakhstan, will be pivotal for reducing the global AMR burden and safeguarding both animal and public health.

## CONCLUSION

This review highlights that AMR in foodborne pathogens, particularly *E. coli* and *Salmonella* spp., poses an escalating threat to both public and animal health. The synthesis of global evidence underscores that foods of animal origin serve as critical reservoirs and transmission pathways of resistant bacteria along the farm-to-fork continuum. High-income regions have demonstrated measurable success in AMR containment through harmonized surveillance and strict AMS, whereas low- and middle-income countries, including Kazakhstan, continue to face challenges such as unregulated AMU, limited diagnostic capacity, and fragmented data systems.

Practical implications of this review emphasize the urgent need for context-specific interventions: strengthening surveillance networks, enforcing veterinary drug regulations, and promoting responsible antibiotic use through AMS programs. Moreover, alternative approaches, such as bacteriophage therapy, probiotics, phytogenic additives, and vaccination, offer promising adjuncts to reduce antimicrobial dependence in livestock production.

The key strength of this review lies in its integrated One Health perspective, bridging data from animal, human, and environmental domains while highlighting the unique gaps in Central Asia and other underrepresented regions. However, limitations persist due to the scarcity of standardized surveillance data, regional variability in reporting quality, and limited field validation of emerging alternative therapies.

Future research should prioritize genomic surveillance, economic impact assessments, and pilot-scale trials for non-antibiotic interventions under real production conditions. Developing harmonized monitoring systems and expanding local research infrastructure will be crucial for sustainable progress.

In conclusion, addressing AMR in foodborne pathogens requires a synergistic approach, combining surveillance, stewardship, and innovation within a unified One Health framework. Only through global collaboration, equitable resource allocation, and region-specific implementation can we ensure safe food systems and safeguard the efficacy of antimicrobials for future generations.

## AUTHOR’S CONTRIBUTIONS

LDZ: Conceptualization, methodology, writing, data collection, analysis, review, and editing. The author has read and approved the final manuscript.
